# The relationship between mental health and risky decision-making in children and adolescents: a scoping review

**DOI:** 10.1186/s12888-024-05850-9

**Published:** 2024-06-05

**Authors:** Francesca Bentivegna, Efstathios Papachristou, Eirini Flouri

**Affiliations:** Department of Psychology and Human Development, UCL Institute of Education, University College London, 25 Woburn Square, London, WC1H 0AA UK

**Keywords:** Adolescence, Decision-making, Externalizing symptoms, Gambling task, Internalizing symptoms, Reward processing

## Abstract

**Background:**

Evidence from studies on adult participants and clinical samples of children suggest an association between risky decision-making and mental health problems. However, the extent and nature of this association in the general youth population remains unknown. Therefore, this scoping review explores the current evidence on the relationship between mental health (internalising and externalising symptoms) and risky decision-making in the general youth population.

**Methods:**

A three-step search strategy was followed and applied to four databases. Selection criteria included participants < 18 years representative of the general population, and information on both risky decision-making (assessed using gambling tasks) and internalising /externalising symptoms. Data were extracted and synthesised for study and participant characteristics, aspects and measures for the main variables, and key findings.

**Results:**

Following screening, twenty-one studies were retrieved. Non-significant associations were more frequent than significant associations for both internalising and externalising symptoms, particularly for social difficulties and broad externalising symptoms. Among the significant associations, hyperactivity/inattention and conduct problems appeared to be positively associated with risk-taking and negatively associated with quality of decision-making. However, patterns were less clear for links between risky decision-making and internalising symptoms, especially between risk-taking and anxiety symptoms.

**Conclusions:**

The present review suggests predominantly a lack of relationship between risky decision-making and mental health problems, and outlines several possible reasons for it. However, when specificity is considered carefully there seems to be a link between risk-taking and specific externalising problems. Future research should employ study designs aimed at disentangling the direction of this relationship and identifying specific aspects of mental health and risky decision-making that could be eventually addressed by tailored interventions.

**Supplementary Information:**

The online version contains supplementary material available at 10.1186/s12888-024-05850-9.

## Background

Risky decision-making is defined as “the process of choosing between competing courses of actions” when “the outcomes of decisions we make are uncertain and associated with the possibility of leading to undesirable results, therefore they involve taking risks” ([1] pp180). As a consequence, risk-taking is an integral part of risky decision-making. Given that making decisions under uncertainty involves pondering both benefits and risks, risky decision-making is also linked to reward and punishment sensitivity, i.e., the extent to which one’s actions are driven by one’s approach to reward (gains) and punishment (losses) [2, 3]. In fact, neurobiological evidence suggests that adolescent risky decision-making is associated with increased activation in reward-related brain regions, such as the ventral medial prefrontal cortex and ventral striatum [4].

There is also much evidence to show that risky decision-making in adolescence is associated with a number of mental health disorders [5], including attention-deficit/hyperactivity disorder (ADHD) [6, 7], antisocial disorder [8], depression [5, 9], anxiety [10], schizophrenia [11], substance abuse [12] and eating disorders [13]. Nonetheless, a review by Sonuga-Barke et al. (2016) that focused on the relationship of decision-making with internalising (depression and anxiety) and externalising (ADHD and conduct disorder) disorders identified two important knowledge gaps: the developmental unfolding of risky decision-making, and the direction of its association with youth mental health [5].

Given that decision-making is a strategic process of choice under risk where an assessment of costs and benefits, both in the short- and in the long-term, takes place, one of the most common and effective ways to measure risky decision-making is through the use of gambling tasks [14–16]. For instance, the Cambridge Gambling Task (CGT) [16] is used to assess various aspects of decision-making, including the ability of adjusting the decision depending on the likelihood of winning. For this reason, gambling tasks are particularly suitable to measure not only risk-taking, but also other related aspects of risky decision-making, such as the time taken to make a choice. Contrary to other reinforcement learning tasks, the CGT and other popular gambling tasks, such as the Iowa Gambling Task (IGT) [17] and the Balloon Analogue Risk Task (BART) [18] assess decision-making under uncertainty, thus modelling “real life” decision-making and conferring ecological validity.

However, most studies on risky-decision making and mental health are cross-sectional or based on adult samples or clinical child samples [5, 19]. Hence, it is not clear what the association is between specific mental health symptoms, such as internalising and externalising problems, and risky decision-making in the general youth population. Research in general population samples is particularly relevant as symptoms can be debilitating despite not reaching the clinical threshold for a diagnosis. Furthermore, examining these symptoms before they become clinically significant can help understand how these may develop and worsen over time. A focus on childhood and adolescence therefore is key, given that mental health problems tend to emerge then [20, 21]. It is also not clear whether findings from studies using clinical samples completing gambling tasks are replicable in this population [5, 10]. Due to the existence of different types of gambling tasks (including different versions of the same tasks adapted for different age groups) measuring different aspects of reward processing, a more in-depth exploration of the evidence on the relationship between mental health and risky decision-making, as measured by these tasks, is also needed. For instance, adaptations of the IGT have been made to allow the evaluation of decision-making in children, where the reward is represented by points or stickers rather than money, or where the cards of the decks show animals instead of letters. In many cases, in the child and adolescent versions, instructions are simplified and the number of trials is lower. What is more, children and adolescents seem to process rewards and make risky decisions differently from adults [22, 23]. For instance, one study evaluated the performance on the IGT of individuals aged 5 to 89 years and found that both the strategic judgement and the cognitive ability displayed by children were different from those found in young and older adults, in turn explaining differences in children’s decision-making performance [24].

Therefore, the objectives of this review are to: i) explore the breadth of evidence on the relationship between internalising and externalising symptoms and risky decision-making (measured using gambling tasks) in childhood and adolescence; ii) identify the main aspects of these mental health problems that are associated with risky decision-making, as well as the direction of these relationships (i.e., whether risky decision-making predicts or is predicted by mental health problems); and iii) map and summarise the available evidence on these relationships in order to inform and identify priorities for future research on this topic.

## Methods

The protocol for this scoping review, which was updated prior to the beginning of the search for the current review, was registered on Open Science Framework (Registration 10.17605/OSF.IO/N293C) and reported in line with the PRISMA-ScR guidelines for the reporting of scoping reviews (see Supplementary material). [Note: The title of the protocol differs from the title of the present article in that we deemed “risky decision-making” a better conceptualisation compared to “reward processing”, which, depending on the definition, might encapsulate decision-making aspects other than the ones recorded by gambling tasks. Nonetheless, we appreciate that some of the authors of the included studies used “reward processing” to describe the outcome measures of the gabling task].

### Search method

We referred to the three-step search strategy proposed by the Joanna Briggs Institute in their Manual for Evidence Synthesis (https://synthesismanual.jbi.global) [25]. We deemed one reviewer (F.B.) to be sufficient to carry out these steps as each step was thoroughly discussed in team meetings and approved by the team. First, a search was conducted on two databases only, Medline (Ovid) and Scopus, to identify all the relevant keywords and index terms (see Supplementary material for the included keywords/index terms). Titles and abstracts of the first 25 retrieved papers for each database were analysed and discussed with the research team. Second, the main search was updated and extended to two more databases, Embase (Ovid) and PsycINFO (Ovid). Searches were conducted from study inception to April 2022. Third, hand-searching of the reference lists of the selected papers was conducted to ensure that all the key papers were included. Any discrepancies against the selection criteria during the full-text screening were solved through team meetings until overall consensus was reached. The authors of the retrieved studies with no full-texts available were contacted to provide them. The search strategy used for Medline is in the Supplementary material. Mendeley Reference Management (https://www.mendeley.com/), Zotero (https://www.zotero.org/) and Covidence (http://www.covidence.org/) were used to store citations and full-text of the papers.

### Selection criteria

Studies were included if the samples comprised children and/or adolescents (< 18 years) representative of the general, non-clinical population, and if internalising and externalising symptoms were measured with quantitative tools, e.g., via questionnaires. Studies were excluded if participants were not recruited from the general population (e.g., patients, high-risk samples, healthy matched samples specifically selected because they did not have one or more symptoms/disorders).

We included studies that investigated the associations of aspects of risky decision-making (e.g., decision-making quality, risk-taking) measured using gambling tasks, and mental health problems. Our definition of mental health included a) internalising (affective) symptoms, e.g., anxiety and depressive symptoms, and peer-relationship problems; and b) externalising (behavioural) symptoms, e.g., hyperactivity/inattention, and antisocial and conduct problems. We accept that concepts such as impulsivity or sensation-seeking are behaviours or traits that can be conceptually described as externalising symptoms. Nonetheless, those terms are more widely used to describe aberrant decision-making. To be able to fully differentiate between externalising symptoms and risky decision-making, we decided to only include papers that clearly used those terms (e.g., risk-taking and sensation-seeking) to describe risky decision-making and not externalising symptoms. Finally, we decided to exclude other mental health problems such as psychotic symptoms, substance abuse and eating disorders, because their relationships with reward processing is well-established [26–29]. In particular, two meta-analyses provide evidence for the relationship of eating disorders and addictive disorders with dysfunctional or impaired decision-making [30, 31]. Moreover, for this particular review we were interested in both childhood and adolescence as key periods for the development of mental health problems, whereas the aforementioned disorders tend to emerge in adolescence.

Only articles written in English were included. We did not apply any limits related to the country where the studies had been conducted. We included observational (cross-sectional, longitudinal) studies, and experimental studies where applicable. Systematic reviews and meta-analyses were excluded. However, they were separately searched and used to inform the search strategy and refine the variable definitions. Only peer-reviewed studies were included. In terms of statistical analyses, in the presence of interaction terms, we only considered the main (direct) effect of risky decision-making on mental health (and vice versa), i.e., we excluded moderation and interaction effects. The findings were considered to be significant when *p*-values < 0.05.

### Data extraction and synthesis

Extracted information included study characteristics (author(s), year of publication, country of origin, study design, time between baseline and latest follow-up, sample size), participants’ characteristics (age, sex), variables measured (exposures/outcomes, type of measure, confounders/covariates), and key findings (direction of significant findings, whether associations were positive/negative, estimates of effect for main results).

**Fig. 1:**
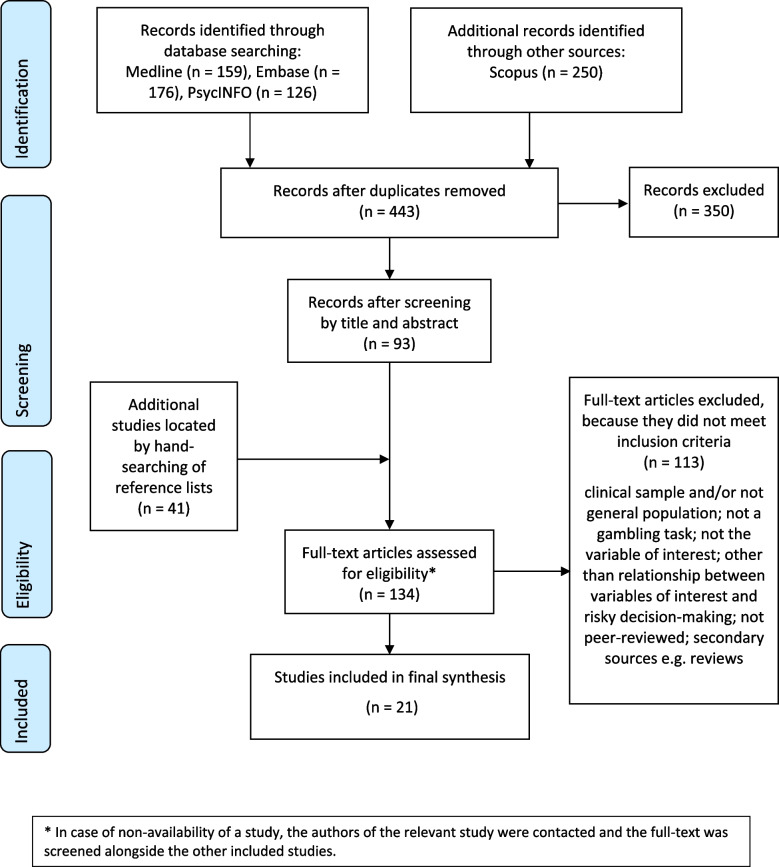
PRISMA flow diagram of the included studies (association between risky decision-making and internalising/externalising symptoms)

We used the PRISMA-ScR flowchart to illustrate the different stages of the search strategy as well as the number of papers retrieved at each stage (see Fig. 1). Following data extraction, we descriptively mapped out the main findings by providing key summary statistics. Specifically, we first summarised study and population characteristics and the specific constructs used to get an overview of the included studies. Next, we provided descriptions and frequencies of the different gambling tasks used, and of the internalising and externalising symptoms investigated and their measurement, paying particular attention to the different domains of internalising and externalising symptoms. Then, we addressed the research question by looking at the significance of the associations and their direction depending on the study design used and the different types of adjustment applied. Effect sizes were included when applicable. The findings and their impact were interpreted in the context of the study design, the developmental phase and sex of the children or adolescents, the confounding variables included in the analyses, and the type of measurement used.

## Results

### Characteristics of included studies

The literature search strategy is summarised in the flowchart (Fig. 1). After removing duplicates, 443 studies were retrieved, of which 350 were excluded against the eligibility criteria. The full-texts of 134 studies (including four studies that were retrieved after contacting the authors) were screened with 20 studies being eligible for inclusion in this review. One more paper was retrieved from the hand-search of the reference lists, bringing the total to 21 studies. The characteristics of the included studies are displayed in Table 1. The included studies employed either a longitudinal (*n* = 8) or a cross-sectional design (*n* = 13), with some of these using an experimental design (including a quasi-experimental design). Most studies were located in the UK (*n* = 8), followed by the US (*n* = 5), the Netherlands (*n* = 3), Australia (*n* = 2), Canada (*n* = 1), China (*n* = 1), and Germany (*n* = 1). Almost half of the studies recruited samples of children (0–12 years; *n* = 10), a quarter focused on adolescents (13–18 years; *n* = 5) and approximately a third covered both developmental phases (*n* = 6). All studies used mixed-sex samples where the ratio female-male was approximately equal. The smallest sample size was *n* = 34 and the largest was *n* = 17,160. The total number of participants in observational studies was *n* = 64,076, whereas in experimental studies it was *n* = 265. Among the longitudinal studies, the follow-up time from baseline to the latest follow-up ranged 3–9 years.

**Table 1:** Characteristics of included studies assessing the associations between risky decision-making and internalising and externalising symptoms

*Study*	*Country*	*Study design*	*Sample size*	*Age*	*% females*	*Risky decision-making aspect(s)*	*Gambling task*	*IE symptom(s)*	*IE measure(s)*
Brandt et al. 2019 [41]	UK	Cross-sectional	1,280	14 years	46.6	Risk taking, quality of decision making, proportional size of the bet participants placed	CGT	Non-obscene socially inappropriate behaviours (being rude/noisy, misbehaving in lessons)	Frequency of behaviours
Bubier & Drabick, 2008 [42]	US (Philadelphia)	Cross-sectional	63	7–9 years	46	Affective decision-making	Children’s Gambling Task	ADHD and ODD symptoms	Child Symptom Inventory-4 (CSI-4)
Flouri & Papachristou, 2019 [54]	UK	Longitudinal	13,888	11–14 years (sweep 5 + sweep 6)	49.4	Decision-making (ages 11 and 14)	CGT	Peer problems (ages 11 and 14)	SDQ
Flouri et al. 2018 [55]	UK	Longitudinal	16,844	3–11 years	49	Decision-making (age 11): ﻿risk taking, quality of decision-making, deliberation time, risk adjustment, delay aversion and overall proportion bet	CGT	Internalising and externalising problems	SDQ
Flouri et al. 2017 [39]	UK	Longitudinal	17,160	3–11 years	49.2	Risk taking, quality of decision making	CGT	Emotional problems, conduct problems, hyperactivity, peer problems	SDQ
Garon & English, 2021 [34]	Eastern Canada	Cross-sectional	86 (41 3-year olds + 45 4-year olds)	3–4 years	61 (3-year olds); 33.3 (4-year olds)	Decision-making	PGT	Peer relationship problems	SDQ
Hooper et al. 2008 [47]	US (Minnesota)	Cross-sectional	153	9–17 years	56.2	Decision-making	IGT	Externalising problems	Achenbach Child Behaviour Checklist
Kirsch & Windmann, 2009 [51]	Germany	Cross-sectional	67	7–14 years	49.3	Decision-making	IGT (two different versions)	Anxiety levels, risk aversion (intolerance towards uncertainty)	Kinder Angst Test II (KAT II)
Lewis et al. 2021 [53]	UK	Longitudinal	10,396 (cross-sectional); 8,628 (longitudinal)	11–14 years (sweep 5 + sweep 6)	50 (cross-sectional); 50 (longitudinal)	Risk-taking, risk adjustment (ages 11 and 14)	CGT	Child emotional symptoms (cross-sectional); Adolescent depressive symptoms (longitudinal)	SDQ; sMFQ
Loheide-Niesmann et al. 2021 [56]	The Netherlands	Longitudinal	875	11–12 years	49.1	Risk-taking behaviour	BART	Internalising behaviour	Substance Use Risk Profile Scale (SURPS)
Loman et al. 2014 [44]	US (Minneapolis)	Cross-sectional	58	M age: 13.0 (0.8)	48.3	Risk-taking	BART-Y	Conduct problems, depressive symptoms	Adolescent Behavior Assessment System for Children, Second edition (BASC-2)
McKewen et al. 2019 [57]	Australia	Cross-sectional	62	15–18 years	53.2	Delay aversion, risk adjustment (impulsive decisions)	CGT	Psychological distress	Depression Anxiety Stress Scale (DASS)
O’Toole et al. 2017 [43]	UK	Cross-sectional	106	3.8 (46 months)-6.7 years (80 months)	51.9	Affective decision-making (hot executive functioning EF)	Children’s Gambling Task	Physical aggression, relational aggression	Preschool Proactive and Reactive Aggression Scale
Poon, 2018 [45]	China	Cross-sectional	136	12–17 years	52.2	Delay aversion, risk adjustment	CGT	Emotional problems, conduct problems, hyperactivity, peer problems	SDQ
Reynolds et al. 2013 [50]	US (Washington)	Quasi-experimental	34 (18 low social anxiety, 16 high social anxiety)	15–18 years	67.6	Risk-taking behaviour	BART	Social anxiety	Abbreviated Social Phobia and Anxiety Inventory (SPAI-23)
Rice et al. 2015 [52]	UK	Non-randomised longitudinal	256 (236 completing pre-assessments; 227 completing post-assessments)	13–14 years	48.4	Reward-processing	CGT (shortened version)	Depressive symptoms; Negative self-beliefs	sMFQ; Dysfunctional Attitudes Scale for Children (DASC)
Romer et al. 2009 [48]	US (Philadelphia)	Cross-sectional	387	10–12 years	51	Reward processing	BART	Internalising symptoms, externalising symptoms	Youth Self Report of the Achenbach System of Empirically Based Assessment
Sheffield et al. 2015 [35]	UK	Cross-sectional	78	11–18 years	49.5 (from recruited sample = 105)	Reward and punishment	Money Maker Task	Externalising behaviour	Externalizing Disorder Inventory (EDI)
Tieskens et al. 2018 [40]	The Netherlands	Longitudinal	1086	7–11 years	49	Risk-taking	BART	Aggression, antisocial and oppositional defiant behaviour	Peer-nominations (2 questions, peer reports); Problem Behaviour at School Interview (short version, teacher reports)
Tieskens et al. 2021 [49]	The Netherlands	Longitudinal	1200	8–12 years	50	Risk-taking	BART-Y	Anxiety symptoms	Revised Child Anxiety and Depression Scale, Peer reports of anxiety symptoms (peer reports), Problem Behaviour at School Interview (short version, teacher reports)
Wilson et al. 2022 [46]	Australia	Cross-sectional	126	5–12 years	53.2	Risk adjustment	CGT	Conduct problems	SDQ

*IE* Internalising/externalising symptoms, *CGT* Cambridge Gambling Task, *PGT* Preschool Gambling Tasks, *IGT* Iowa Gambling Task, *BART* Balloon Analogue Risk Task, *BART-Y* Balloon Analogue Risk Task-Youth, *SDQ* Strengths and Difficulties Questionnaire, *sMFQ* Short Mood and Feelings Questionnaire

### Gambling task types

Table S1 in the Supplementary material describes all the gambling tasks used in the included studies, and their different versions if applicable. As seen in Table S2, the main gambling tasks used to assess risky decision-making (often defined as decision-making or risk-taking in the included studies, as previously mentioned) in the context of internalising and externalising symptoms were the CGT (*n* = 9), followed by the BART (*n* = 4) and the version of the BART used in youth (BART-Y; *n* = 2) [32]; the IGT (*n* = 2) and two versions of the task modified for children, the Children’s Gambling Task (*n* = 2) [33] and the Preschool Gambling Task (PGT; *n* = 1) [34]; and the Money Maker Task (*n* = 1) [35]. The Children’s Gambling Task and the PGT were developed as card games to make the task more “child-friendly”, whereas all the other measures were computerised assessments.

### Internalising and externalising symptoms

Approximately one fourth of the studies focused on both internalising and externalising symptoms (*n* = 5). Among the rest, there was a roughly equal split of studies focussing on internalising (*n* = 9) or externalising symptoms only (*n* = 7). The main internalising symptoms were emotional problems, including depressive and anxiety symptoms (*n* = 11), and the rest of the studies focused on peer-relationship problems (*n* = 4). The main externalising symptoms were conduct problems, including aggressive, antisocial or inappropriate behaviours (*n* = 9), and hyperactivity/inattention symptoms (*n* = 3).

Internalising and externalising symptoms were measured via several self-, parent-, or teacher-reported scales (see Table S2 in the Supplementary material). The Strengths and Difficulties Questionnaire (SDQ) [36] was the most frequently used measure (*n* = 6), followed by the short Mood and Feelings Questionnaire (*n* = 2) [37], the short version of the Problem Behaviour at School Interview (*n* = 2) [38], and peer-reports of anxiety (*n* = 2). All other measures were used in one study each.

### Association of internalising and externalising symptoms with risky decision-making domains

Table 2 illustrates the frequency and overall significance and direction of the individual associations found in each study stratified by symptom domain (internalising and externalising) and study design. Details of the specific associations are reported in Tables 3 and 4, including direction and significance. Most longitudinal studies (Table 3) investigated this association using internalising and/or externalising symptoms as the outcome (*n* = 4), one study used risky decision-making as the outcome, and two studies explored the reciprocal associations between internalising/externalising symptoms and risky decision-making. Table 4 shows the individual associations as well as the covariates/confounders used in each cross-sectional and quasi-experimental study, respectively.

**Table 2:** Frequency and significance of associations with specific domains of risky decision-making by specific internalising/externalising symptoms

			*Significance – N main individual associations* + *specific risky decision-making domain*		
	*Specific mental health domain*	*Study design* *(N studies)*	*Positive association*	*Negative association*	*Not significant*
**Internalising/ externalising symptoms**	Emotional problems (e.g. depressive symptoms, anxiety)	Longitudinal (5)	/	1 (CGT risk-taking); 7 (BART-Y risk-taking); 1 (CGT reward seeking)	6 (CGT risk-taking); 1 (CGT quality of decision-making); 6 (CGT risk adjustment); 2 (BART risk-taking behaviour); 5 (BART-Y risk-taking)
		Cross-sectional (4)	1 (IGT shuffled decision-making performance)	1 (CGT risk adjustment)	1 (IGT original overall decision-making performance); 1 (BART-Y risk-taking); 2 (CGT delay aversion); 1 (CGT risk adjustment)
		Quasi-experimental (1)	1 (BART risk-taking)	/	/
	Peer problems	Longitudinal (2)	/	/	5 (CGT risk-taking); 4 (CGT risk adjustment); 5 (CGT quality of decision-making); 4 (CGT delay aversion); 4 (CGT deliberation time)
		Cross-sectional (2)	/	1 (PGT decision-making strategies/adaptation)	1 (CGT risk adjustment); 1 (CGT delay aversion)
	Conduct problems (e.g. aggressive, antisocial or inappropriate behaviours)	Longitudinal (2)	1 (CGT risk-taking); 3 (BART risk-taking)	1 (CGT quality of decision-making)	7 (BART risk-taking)
		Cross-sectional (6)	1 (CGT risk-taking); 1 (CGT proportional size bet)	1 (CGT quality of decision-making)	1 (CGT risk-taking); 1 (CGT proportional size bet); 1 (quality of decision-making); 2 (Children’s Gambling Task proportion of advantageous choices); 1 (BART-Y risk-taking); 2 (Children’s Gambling Task affective decision-making); 2 (CGT risk adjustment); 1 (CGT delay aversion)
	Hyperactivity/inattention	Longitudinal (1)	1 (CGT risk-taking)	1 (CGT quality of decision-making)	/
		Cross-sectional (2)	/	1 (Children’s Gambling Task proportion of advantageous choices)	3 (Children’s Gambling Task proportion of advantageous choices); 1 (CGT risk adjustment); 1 (CGT delay aversion)
	General internalising symptoms	Cross-sectional (1)	/	1 (BART reward processing)	/
	General externalising symptoms	Cross-sectional (3)	/	/	1 (IGT decision-making performance); 1 (BART reward processing); 2 (Money Maker Task punishment sensitivity/reward sensitivity)
	Both internalising and externalising symptoms^a^	Longitudinal (1)	2 (CGT delay aversion); 1 (CGT risk-taking); 1 (CGT deliberation time); 1 (CGT overall proportional bet)	3 (CGT quality of decision-making); 3 (CGT risk adjustment); 1 (CGT delay aversion)	3 (CGT risk-taking); 1 (CGT delay aversion); 3 (CGT deliberation time); 3 (CGT overall proportional bet); 1 (CGT quality of decision-making); 1 (CGT risk adjustment)

*CGT* Cambridge Gambling Task, *PGT* Preschool Gambling Task, *IGT* Iowa Gambling Task, *BART* Balloon Analogue Risk Task, *BART-Y* Balloon Analogue Risk Task-Youth

^a^This domain refers specifically to a paper (Flouri et al. 2018) where children were categorised according to whether they presented with steadily increasing internalising and externalising problems (‘deteriorators’) or high levels of internalising and externalising problems and low IQ (‘troubled’)

**Table 3:** Longitudinal studies (*n* = 8) – Risky decision-making and internalising/externalising symptoms (direction and significance)

*Study*	*Risky decision-making*	*Internalising/ externalising (IE) symptoms*	*Developmental phase (time between baseline and last follow-up)*	*Controlled for*	*Direction & significance of adjusted (wherever relevant) associations*
Flouri & Papachristou, 2019 [54]	CGT Decision-making	Peer problems	Childhood/adolescence (3 years)	Maternal age, maternal education, emotional and behavioural problems, ethnicity, exact age at assessment, poverty (number of sweeps below the poverty line, defined as 60% of the UK median household income1), and intelligence. Interaction/controlled for: Bullying. Analyses stratified by sex	Males: - CGT Risk-taking → Peer problems (n.s.) - CGT Risk adjustment → Peer problems (n.s.) - CGT Quality of decision-making → Peer problems (n.s.) - CGT Delay aversion → Peer problems (n.s.) - CGT Deliberation time → Peer problems (n.s.) - Peer problems → CGT Risk-taking (n.s.) - Peer problems → CGT Risk adjustment (n.s.) - Peer problems → CGT Quality of decision-making (n.s.) - Peer problems → CGT Delay aversion (n.s.) - Peer problems → CGT Deliberation time (n.s.) Females: - CGT Risk-taking → Peer problems (n.s.) - CGT Risk adjustment → Peer problems (n.s.) - CGT Quality of decision-making → Peer problems (n.s.) - CGT Delay aversion → Peer problems (n.s.) - CGT Deliberation time → Peer problems (n.s.) - Peer problems → CGT Risk-taking (n.s.) - Peer problems → CGT Risk adjustment (n.s.) - Peer problems → CGT Quality of decision-making (n.s.) - Peer problems → CGT Delay aversion (n.s.) - Peer problems → CGT Deliberation time (n.s.)
Flouri et al. 2018 [55]	CGT Decision-making	IE problems (emotional and peer problems; hyperactivity and conduct problems)	Childhood (8 years)	Child’s birth weight, breastfeeding status and ethnicity, maternal education, maternal smoking status, maternal age at child’s birth, maternal psychological distress, family structure and socioeconomic disadvantage; household chaos, parent–child relationship, parental involvement, quality of emotional support, harsh parental discipline and regular bedtimes. Analyses stratified by sex	Males: - High IE problems → CGT Risk-taking (n.s.) - **High IE problems ** →** CGT Delay aversion (positive)** - High IE problems → CGT Deliberation time (n.s.) - High IE problems → CGT Overall proportional bet (n.s.) - **High IE problems ** →** CGT Quality of decision-making (negative)** - **High IE problems ** →** CGT Risk adjustment (negative)** - Steadily increasing IE problems → CGT Risk-taking (n.s.) - Steadily increasing IE problems → CGT Delay aversion (n.s.) - Steadily increasing IE problems → CGT Deliberation time (n.s.) - Steadily increasing IE problems → CGT Overall proportional bet (n.s.) - Steadily increasing IE problems → CGT Quality of decision-making (n.s.) - Steadily increasing IE problems → CGT Risk adjustment (n.s.) Females: - High IE problems → CGT Risk-taking (n.s.) - High IE problems → CGT Delay aversion (n.s.) - **High IE problems ** →** CGT Deliberation time (positive)** - High IE problems → CGT Overall proportional bet (n.s.) - **High IE problems ** →** CGT Quality of decision-making (negative)** - **High IE problems ** →** CGT Risk adjustment (negative)** - **Steadily increasing IE problems ** →** CGT Risk-taking (positive)** - **Steadily increasing IE problems ** →** CGT Delay aversion (positive)** - **Steadily increasing IE problems ** →** CGT Deliberation time (positive)** - **Steadily increasing IE problems ** →** CGT Overall proportional bet (positive)** - **Steadily increasing IE problems ** →** CGT Quality of decision-making (negative)** - **Steadily increasing IE problems ** →** CGT Risk adjustment (negative)**
Flouri et al. 2017 [39]	CGT Risk taking, quality of decision making	Emotional problems, conduct problems, hyperactivity, peer problems	Childhood (8 years)	Child's gender, child's ethnicity, maternal education, family structure, family poverty, maternal depression, child’s working memory	- CGT Risk taking → Emotional problems (n.s.) - **CGT Risk taking ** →** Conduct problems (positive)** - **CGT Risk taking ** →** Hyperactivity (positive)** - CGT Risk taking → Peer problems (n.s.) - CGT Quality of decision-making → Emotional problems (n.s.) - **CGT Quality of decision-making ** →** Conduct problems (negative)** - **CGT Quality of decision-making ** →** Hyperactivity (negative)** - CGT Quality of decision-making → Peer problems (n.s.)
Lewis et al. 2021 [53]	CGT Risk-taking, risk adjustment (ages 11 and 14)	Child emotional symptoms (cross-sectional) and adolescent depressive symptoms (longitudinal)	Childhood/adolescence (3 years)	﻿Family income, maternal education, child age at the time of the exposure, ethnic background, stage of pubertal development, child cognitive ability (as a proxy for intelligence quotient), parent depressive symptoms and, in longitudinal analyses, children’s baseline emotional symptoms and behavioural problems. Some analyses adjusted for sex. Some analyses stratified by sex	Cross-sectional results: General: - CGT Risk-taking – Emotional symptoms (n.s.) - CGT Risk adjustment – Emotional symptoms (n.s.) Females: - CGT Risk-taking – Emotional symptoms (n.s.) - CGT Risk adjustment – Emotional symptoms (n.s.) Males: - CGT Risk-taking – Emotional symptoms (n.s.) - CGT Risk adjustment – Emotional symptoms (n.s.) Longitudinal results: General: - CGT Increased risk-taking → Decreased depressive symptoms (n.s.) - CGT Risk adjustment → Depressive symptoms (n.s.) Females: - **CGT Risk-taking ** →** Depressive symptoms (negative)** - CGT Risk adjustment → Depressive symptoms (n.s.) Males: - CGT Risk-taking → Depressive symptoms (n.s.) - CGT Risk adjustment → Depressive symptoms (n.s.)
Loheide-Niesmann et al. 2021 [56]	BART Risk-taking behaviour	Internalising behaviour	Childhood (N/A)	N/A (moderation analyses were adjusted for covariates, but only correlation analyses are relevant to this study)	- Anxiety sensitivity – BART Risk-taking behaviour (n.s.) - Hopelessness – BART Risk-taking behaviour (n.s.)
Rice et al. 2015 [52]	CGT Reward-processing	Depressive symptoms; Negative self-beliefs	Adolescence (9 years)	Model 1 adjusts for gender; age, baseline reward seeking, and quality of decision making. Model 2 adjusts for age, gender, baseline total dysfunctional attitudes score, baseline number of agreements on dysfunctional attitudes scale and baseline reaction time different to agree with versus disagree with dysfunctional attitudes. Model 3 adjusts for age, gender and baseline over-general memory	- **CGT Change (increase) in reward-seeking ** →** Greater change (reduction) in depression (negative inverted)**
Tieskens et al. 2018 [40]	BART Risk-taking	Aggression, antisocial and oppositional defiant behaviour	Childhood (4 years)	Covariate: age. Control (path estimates): Socio-economic status. Multiple group model to test for sex differences	Males: - Peer-reported: - **BART High risk-taking ** →** High aggression (positive)** - BART Risk-taking → High oppositional defiant behaviour (n.s) - Teacher-reported: - BART Risk-taking → Aggression (n.s) - BART Risk-taking → Covert antisocial behaviour (n.s) - BART Risk-taking → Oppositional defiant behaviour (n.s.) Females: - Peer-reported: - **BART High risk-taking ** →** High aggression (positive)** - **BART High risk-taking ** →** High oppositional defiant behaviour (positive)** - Teacher-reported: - BART Risk-taking → Aggression (n.s) - BART Risk-taking → Covert antisocial behaviour (n.s) - BART Risk-taking → Oppositional defiant behaviour (n.s.)
Tieskens et al. 2021 [49]	BART-Y Risk-taking	Anxiety symptoms	Childhood (4 years)	Multiple group comparison to test for sex differences	Males: - Self-reported: - **Increased anxiety symptoms ** →** BART-Y Decreased risk-taking (negative)** - BART-Y Risk-taking → Anxiety (n.s.) - Peer-reported: - **Increased anxiety symptoms ** →** BART-Y Decreased risk-taking (negative)** - **BART-Y Decreased risk-taking ** →** Increased anxiety symptoms (negative inverted)** - Teacher-reported: - Increased anxiety symptoms → BART-Y Decreased risk-taking (n.s.) - BART-Y Risk-taking → Anxiety symptoms (n.s.) Females: - Self-reported: - **Increased anxiety symptoms ** →** BART-Y Decreased risk-taking (negative)** - BART-Y Risk-taking → Anxiety (n.s.) - Peer-reported: - **Increased anxiety symptoms ** →** BART-Y Decreased risk-taking (negative)** - **BART-Y Decreased risk-taking ** →** Increased anxiety symptoms (negative inverted)** - Teacher-reported: - **Increased anxiety symptoms ** →** BART-Y Decreased risk-taking (negative)** - BART-Y Risk-taking → Anxiety symptoms (n.s.)

*CGT* Cambridge Gambling Task, *PGT* Preschool Gambling Task, *IGT* Iowa Gambling Task, *BART* Balloon Analogue Risk Task, *BART-Y* Balloon Analogue Risk Task-Youth, n.s. not significant

**Table 4:** Cross-sectional (*n* = 12) and quasi experimental studies (*n* = 1) – Risky decision-making and internalising/externalising symptoms (direction and significance)

*Study*	*Risky decision-making*	*Internalising/ externalising (IE) symptoms*	*Developmental phase*	*Covariates/confounders*	*Significance of adjusted (wherever relevant) associations*
Cross-sectional studies					
Brandt et al. 2019 [41]	CGT Risk taking, quality of decision-making, proportional size of the bet placed	Non-obscene socially inappropriate behaviours (being rude/noisy, misbehaving in lessons)	Adolescence (age 14)	Control variable: emotional problems, sex	- **CGT Risk taking – misbehaving in lessons (positive)** - **CGT Proportional size of bet – misbehaving in lessons (positive)** - **CGT Quality of decision-making – misbehaving in lessons (negative)** - CGT Risk taking – being rude/noisy (n.s.) - CGT Quality of decision-making – being rude/noisy (n.s.) - CGT Proportional size of the bet placed – being rude/noisy (n.s.)
Bubier & Drabick, 2008 [42]	Children’s Gambling Task Affective decision-making	ADHD and ODD symptoms	Childhood (age 7–9)	Covariates: child age, executive functioning, IQ, family income. Mediators: autonomic nervous system (ANS) activity measured using respiratory sinus arrhythmia (RSA) and pre-ejection period (PEP). Analyses stratified by sex	- **Children’s Gambling Task Proportion of advantageous choices – ADHD-H (boys; negative)** - Children’s Gambling Task Proportion of advantageous choices – ADHD-H (girls; n.s.) - Children’s Gambling Task Proportion of advantageous choices – ODD symptoms (boys; n.s.) - Children’s Gambling Task Proportion of advantageous choices – ODD symptoms (girls; n.s.) - Children’s Gambling Task Proportion of advantageous choices – ADHD-I (boys; n.s.) - Children’s Gambling Task Proportion of advantageous choices – ADHD-I (girls; n.s.)
Garon & English, 2021 [34]	PGT Decision-making	Peer relationship problems	Childhood (age 3–4)	Between-subject variable: age (gender was not significant in any of the analyses, hence was removed)	- **Peer problems – PGT Decision-making strategies/adaptation (negative)**
Hooper et al. 2008 [47]	IGT Decision-making	Externalising problems	Childhood/ Adolescence (age 9–17)	Covariates: age and full-scale IQ. Interaction: personality (extraversion and neuroticism)	- Externalising problems – IGT Decision-making performance (n.s.)
Kirsch & Windmann, 2009 [51]	IGT Decision-making	Anxiety levels, risk aversion (intolerance towards uncertainty)	Childhood/ Adolescence (age 7–14)	Confounders: age, depression, gender. Covariates: personality traits, intellectual abilities	- **Anxiety – IGT Shuffled decision-making performance**^**a**^ **(positive)** - Anxiety – IGT Original overall decision-making performance (n.s.)
Loman et al. 2014 [44]	BART-Y Risk-taking	Conduct problems, depressive symptoms	Adolescence (median age 13.0)	N/A (correlation analyses only)	- Conduct problems – BART-Y Risk-taking (n.s.) - Depressive symptoms – BART-Y Risk-taking (n.s.)
McKewen et al. 2019 [57]	CGT Delay aversion, risk adjustment (impulsive decisions)	Psychological distress	Adolescence (age 15–18)	N/A (uncorrected correlation analyses only)	- CGT Delay aversion – Psychological distress (n.s.) - CGT Risk adjustment – Psychological distress (n.s.)
O’Toole et al. 2017 [43]	Children’s Gambling Task Affective decision-making (hot executive functioning EF)	Physical aggression, relational aggression	Childhood (age 3.8–6.7)	Confounders: ﻿child age, gender and verbal ability	- Physical aggression – Children’s Gambling Task Affective decision-making (n.s.) - Relational aggression – Children’s Gambling Task Affective decision-making (n.s.)
Poon, 2018 [45]	CGT Delay aversion, risk adjustment	Emotional problems, conduct problems, hyperactivity, peer problems	Childhood/ Adolescence (age 12–17)	Control variable: general intellectual ability	- **CGT Risk adjustment – Emotional problems (negative inverted)** - CGT Risk adjustment – Conduct problems (n.s.) - CGT Risk adjustment – Hyperactivity (n.s.) - CGT Risk adjustment – Peer problems (n.s.) - CGT Delay aversion – Emotional problems (n.s.) - CGT Delay aversion – Conduct problems (n.s.) - CGT Delay aversion – Hyperactivity (n.s.) - CGT Delay aversion – Peer problems (n.s.)
Reynolds et al. 2013^b^ [50]	BART Risk-taking behaviour	Social anxiety	Adolescence (age 15–18)	N/A (age, gender and race were not significantly related to the primary dependent variable and were not included in further analyses)	- **High social anxiety – BART Risk-taking (positive)**
Romer et al. 2009 [48]	BART Reward processing	Internalising (anxiety, depression)/ externalising (rule breaking, aggressive behaviour) behaviour problems	Childhood (age 10–12)	N/A	- **BART Reward processing – Internalising problems (negative)** - BART Reward processing – Externalising problems (n.s.)
Sheffield et al. 2015 [35]	Money Maker Task Reward and punishment (feedback-related negativity (FRN), P3b)	Externalising behaviour	Childhood/ adolescence (age 11–18)	Covariate: age (gender was not significant and was not included in further analyses)	- Externalising behaviour – Money Maker Task Punishment sensitivity (n.s.) - Externalising behaviour – Money Maker Task Reward sensitivity (n.s.)
Wilson et al. 2022 [46]	CGT Risk adjustment	Conduct problems	Childhood/ Adolescence (age 5–12)	Covariate: age	- CGT Risk adjustment – Parent-reported conduct problems (n.s.)

*CGT* Cambridge Gambling Task, *PGT* Preschool Gambling Task, *IGT* Iowa Gambling Task, *BART* Balloon Analogue Risk Task, *BART-Y* Balloon Analogue Risk Task-Youth, n.s. not significant

^a^Significant losses occur earlier than in the original version in order to prevent the establishment of initial preferences for disadvantageous decks

^b^Quasi-experimental study

#### Frequency and direction of individual significant associations by type of symptoms

Overall, the majority of findings were non-significant. Specifically, just over one fifth of all individual associations with specific internalising symptoms were significant, while the proportion of significant associations for specific externalising symptoms reached one third. As for internalising and externalising symptoms analysed as a composite construct, half of the individual associations were significant.

Among the significant associations, most of the positive associations were between externalising symptoms and risk-taking, while the majority of the negative associations were between externalising symptoms and quality of decision-making, and between internalising symptoms and risk-taking and risk adjustment. Specifically, two longitudinal studies found that risk-taking positively predicted later conduct problems [39, 40], but for one of these studies evidence of significance was present only for the peer-reported, and not for the teacher-reported, measure. Similarly, one cross-sectional study found a positive association of risk-taking and overall proportional bet with some ‘indicators’ of conduct problems (i.e., misbehaving in class) but not others (i.e., being rude or noisy) [41]. That study also found that better quality of decision-making was inversely associated with the same ‘indicators’ of conduct problems. The effect sizes of the associations in these studies were weak to moderate, e.g., fixed effect estimate –0.391 (SE 0.145) and rho –0.09 (*p* < 0.05) for the associations with quality of decision-making. However, there was also a number of cross-sectional studies that failed to find evidence of an association with conduct problems altogether, including two studies using the Children’s Gambling Task [42, 43], one using the BART-Y [44], and two studies using the CGT to measure risk adjustment [45, 46]. Similar results were found for hyperactivity/inattention, with one longitudinal study showing that risk-taking predicts more hyperactivity/inattention, but also that better quality of decision-making predicts a decrease in hyperactivity/inattention [39]. In the same vein, in another study the proportion of advantageous choices was associated with lower hyperactivity [42] (effect sizes are reported in the subsection considering the adjustment for covariates), however, this was only true for girls, and there was no evidence of an association with inattentive symptoms. Instead, the three studies that looked at general (i.e., composite score) externalising symptoms found no significant associations at all [35, 47, 48].

With regard to internalising symptoms, one longitudinal study exploring the reciprocal association between anxiety symptoms and risk-taking found that increased anxiety symptoms predicted a decrease in risk-taking more frequently than the reverse [49]. In contrast, one quasi-experimental study showed a positive association between social anxiety and risk-taking [50], while a cross-sectional study found that anxiety was positively associated with better decision-making, when this was assessed with the shuffled, not the original, version of the IGT [51]. Of note, not all the studies reported effect sizes, as they instead reported correlations of moderate strength, e.g. anxiety was positively correlated to decision-making performance (*r*  0.440; *p* < 0.05) [51]. As for depressive symptoms, it was found that an increase in reward-seeking was predictive of a reduction in depression [52]. There was also evidence from one recent study that increased risk-taking predicted a reduction in depressive symptoms, but only in females [53]. The same study did not find evidence of association between risk adjustment (also examined) and depressive symptoms. In contrast, another study showed that poor risk adjustment was associated with more emotional problems [45]; however, this was not the case with delay aversion, which was also examined. The effect sizes for the significant associations of decision-making with emotional and depressive symptoms ranged from –1.41 to −0.264 (βs; *p* < 0.05). As for peer-relationship problems, one study showed that children with more peer problems displayed poorer adaptive decision-making [34]. The two aspects of decision-making considered were exploration, i.e. the child explores different options, and exploitation, i.e. the child stays with the most profitable option in order to gain the best reward possible, and effect sizes were *r* –0.26 for exploitation (peer problems were linked to less stay after a win from the advantageous deck) and *r* 0.24 for exploration (peer problems were related to greater exploration of different options; both *p* < 0.05). However, there was no longitudinal evidence of an association between peer problems and any of the CGT outcome measures [39], regardless of whether peer problems were the exposure or the outcome [54]. Only one cross-sectional study has explored general internalising symptoms in relation to risky decision-making and found a negative association between them and reward processing [48]. Finally, the one study [55] that considered internalising and externalising symptoms as a whole found an equal number of significant and non-significant associations (details are reported in the next sub-sections).

#### Individual associations in studies stratified by sex

The covariates for each study and information on whether analyses were stratified by sex/gender are displayed in Tables 3 and 4. Effect sizes are reported in the next sub-section.

In general, some results differed by sex, with six studies stratifying by sex. The results of the analyses on externalising symptoms in males and females showed that increased risk-taking predicted more aggression, while risk-taking also predicted oppositional defiant behaviours, albeit only in females [40]. Moreover, the proportion of advantageous choices in a gambling task was associated with lower hyperactivity symptoms in boys, but not girls [42].

However, results were less clear for internalising symptoms. There was evidence for an inverse reciprocal relationship between risk-taking and anxiety symptoms in both boys and girls, though only when symptoms were peer-reported [49]. Another study found an association between high risk-taking and a decrease in depressive symptoms in females only, but no association between emotional symptoms and risk-taking or risk adjustment in males or females [53]. Additionally, no association in either boys or girls was found for peer problems and later decision-making (risk-taking, risk adjustment, quality of decision-making, delay aversion, or deliberation time) or for decision-making and later peer problems [54].

In the last study [55], children were classified according to their internalising and externalising symptom trajectories (stable-low, decreasing, increasing, stable-high), and positive associations were found for girls (but not boys) between symptoms and delay aversion, risk-taking, deliberation time, and overall proportion bet, while negative associations were evidenced for quality of decision-making and risk adjustment. Additionally, stable-high symptoms were associated, negatively in girls and positively in boys, with delay aversion, while for both sexes a negative association was found between quality of decision-making and risk adjustment.

#### Individual associations and effect sizes by levels of adjustment for covariates

Not all studies adjusted for confounders. Some of them chose not to adjust on the basis of null associations in preliminary analyses, while others carried out only bivariate correlation tests. The studies that did adjust for confounding used different covariates, thus making it difficult to compare results (Tables 3 and 4). Overall, most of the studies that did adjust for confounding included covariates such as the participants’ sex or gender, age, ethnicity, intellectual ability, mental health problems, and parental characteristics including socio-economic status—measured as parental education or income—and mental health problems.

Three studies included age only as a covariate, but only one of these found a significant association: experiencing peer-relationship problems was associated with poor decision-making strategies (correlation reported above) [34]. Instead, for the two studies investigating externalising symptoms, there was no evidence of an association between general externalising problems and reward/punishment sensitivity [35], or between risk adjustment and parent-reported conduct problems [46]. One study [45] controlled for intellectual ability only and found one significant negative association between risk adjustment and emotional problems (β −0.264, t −3.053, *p* < 0.05, R^2 0.10).

Four studies adjusted for sex or gender, age and intellectual ability and/or mental health problems. Three of them focused on different aspects of externalising symptoms; significant associations were found for misbehaving in class (*rho* –0.09 to 0.14, *p* < 0.001) [41], but not for general externalising problems [47], being rude or noisy, or relational and physical aggression [56]. The other study [51] focused on anxiety, which was significantly associated with better performance on a modified version of the IGT (correlation reported above).

In addition to some of the covariates discussed, two studies adjusted for socio-economic status. One [40] found significant associations between risk-taking and peer-reported conduct problems including aggression and oppositional-defiant behaviour (β 0.005 to 0.009, SE 0.002 to 0.004, *p* < 0.05). Instead, risk-taking was not associated with aggression, oppositional-defiant disorder or covert antisocial behaviour when these were reported by teachers rather than peers. The other study [42] found one significant association between poor decision-making and ADHD-hyperactive type (β −0.38, *p* < 0.05), whereas associations for ADHD-inattentive type and oppositional-deficit disorder were non-significant.

Lastly, other studies included additional covariates, such as birth weight and pubertal status, maternal age, breastfeeding status and maternal smoking status. One study found non-significant associations between decision-making and peer problems [54]. Instead, two studies investigating depressive symptoms found significant associations: one showed that an increase in reward-seeking predicted a reduction in depression (β –1.41, SE 0.41, *p* < 0.001) [52], while the other found that risk-taking predicted fewer depressive symptoms, but only in females (unstandardised B −0.31, 95% CI −0.60 to  –0.02, *p* = 0.037) [53]. Two studies looked at both internalising and externalising symptoms. One study [55] found that greater severity in both symptom domains was significantly associated with greater risk-taking, more delay aversion, longer deliberation time, poorer quality of decision-making, less risk adjustment, and greater overall proportion bet (β –0.22 to 0.31, SE 0.01 to 0.13, *p* < 0.05). The second study [39] found significant associations for externalising symptoms, where risk-taking predicted higher levels of conduct problems and hyperactivity/inattention, and quality of decision-making predicted lower levels of externalising problems (fixed effect estimate –0.542 to 0.771, SE 0.144 to 0.222, *p* < 0.05), while no significant associations were found for internalising problems.

As for the studies that did not adjust for any covariates, two of those that found evidence of associations explored the relationship between anxiety and risk-taking. In the first study [49], the effect sizes for the association between higher levels of anxiety symptoms and decreased risk-taking differed depending on whether the symptoms were self-, peer-, or teacher-reported, with standardised coefficients ranging from β −0.03 to −0.10 (*p* < 0.05). Only in the case of peer-reported symptoms was lower risk-taking associated with an increase in anxiety symptoms, and for both boys and girls (βs −0.03 to −0.07, *p* < 0.05). The second study [50] found significant associations between high social anxiety and increased risk-taking behaviour (number of explosions on the BART) in high-stress vs low-stress conditions (low-stress: M(SD) = 7.0 (2.73); high-stress: 8.19 (2.71); F(1, 15) 5.09, *p* = 0.04, *d* −0.44), meaning that experiencing high social anxiety in acute stress conditions leads to increased risk-taking. There was also one study [48] which found a negative correlation between general internalising symptoms and reward processing (*r* –0.154, *p* < 0.01). The other three studies in which no adjustment was made [44, 56, 57] explored the association between risky decision-making and internalising behaviours, with one study also looking at the relationship with conduct problems [44]. None found any significant associations.

## Discussion

This scoping review summarised the current evidence on the relationships between internalising and externalising symptoms and risky decision-making measured using gambling tasks, in children and adolescents from the general population. It appeared that, overall, most associations were non-significant. In instances where there was evidence of significance, more distinct patterns could be identified for externalising problems in relation to risky decision-making.

When looking at the characteristics of the studies, we found that the most heavily-used gambling tasks were computerised assessments such as CGT, BART and IGT, whereas a few studies used adaptations of these tasks. Internalising and externalising symptoms were entirely assessed with questionnaires, and the SDQ was the most frequently used measure. As for the specific domains analysed, the majority of the retrieved studies focused on emotional problems (e.g., depressive/anxiety symptoms) and conduct problems (e.g., aggressive/antisocial behaviours), while risk-taking was by far the most studied risky-decision-making aspect.

With regard to the significance of the specific associations between mental health and risky decision-making, some patterns could be identified despite variations in findings, as discussed. In the case of externalising symptoms, positive associations were found for some risky decision-making domains including risk-taking, delay aversion, deliberation time, and overall proportional bet, whereas negative associations were found for quality of decision-making and proportion of advantageous choices. This is in line with what was reported in the review by Sonuga-Barke et al. (2016) [5], which highlighted associations between decision-making and disorders such as ADHD and conduct disorder/oppositional defiant disorder in clinical samples of children and/or adolescents. As for internalising symptoms, our review showed that they were negatively associated with quality of decision-making, risk adjustment, and general reward-seeking. For example, reward-seeking predicted a reduction in depression. This is in line with a review summarising evidence from a clinical sample of adolescents that suggests that the response to reward could be an endophenotype of major depression in adolescence [58]. We note however that in our review the links between internalising symptoms and risk-taking were sometimes positive and sometimes negative. Given that individuals with anxiety are generally deemed to be risk-averse [5], the positive direction of some of the results may appear to be counterintuitive. However, the only study [50] that found a positive association with risk-taking looked at the link with social anxiety. In that study, those with high social anxiety showed more risk-taking, but this might be because they were tested under a stress-inducing condition, hence potentially causing them to take more risky decisions on the BART. This difference in patterns in depression and anxiety symptoms highlights the importance of considering these two constructs separately. For instance, a paper reviewing the literature on anxiety in childhood suggested a developmental model whereby children with anxiety develop depression in adolescence, with one of the proposed vulnerability mechanisms underlying this relationship being reward processing [59]. A more careful consideration and distinction among these constructs is therefore needed to be able to fully examine the type of role that risky decision-making may play in depressive and anxiety symptoms.

This review also identified a rather large number of non-significant relationships. In particular, with one exception [34], peer problems were not significantly associated with decision-making. General externalising symptoms were also not related to risky decision-making [35, 47, 48]. In general, both emotional and conduct problems were more often than not unrelated to risky decision-making, whereas hyperactivity tended to show some links. However, the general lack of significant associations in most studies requires that we consider the possibility that we currently do not have enough evidence to claim the existence of a robust association between risky decision-making and internalising and externalising symptoms in the general youth population.

That being said, there might also be a number of other reasons for the uncertainty around both the direction of the relationships, especially regarding internalising symptoms, and the higher number of non-significant compared to significant associations. To start with, there were stark variations in study design, which likely played a role in the differences in effect sizes. While longitudinal studies in this context are preferrable as they enable the examination of how decision-making and mental health issues evolve over time [5], it should also be acknowledged that effect sizes from studies using a longitudinal design will likely be smaller than those found in cross-sectional [60] and experimental studies. Moreover, the likelihood of finding significant associations also decreases, particularly for those studies lacking the power to detect small effect sizes, hence the comparison among different results becomes more difficult. Another element that might have had an impact on the high variability of the results is that the developmental phase we considered was a wide age range. This becomes problematic as associations might be present only during specific developmental stages (e.g., in childhood but not adolescence). Relatedly, some studies used the original tasks and others the youth-adapted versions of these tasks, which also might have contributed to the differences in results. However, given that the youth-adapted tasks are validated measures [32, 33], it is plausible that they could better identify decision-making aspects in our study population. In a similar vein, sex differences were not explicitly considered in all studies, hence some uncertainty in the findings could be because some associations were sex-specific. For instance, externalising symptoms are usually more common in boys than in girls, but the association with risky decision-making might not be picked up without considering the two sexes separately. Nevertheless, among studies that did stratify analyses by sex, it was not possible to identify a clear pattern of differences in the associations found between males and females. The results also varied considerably depending on which confounders and covariates were controlled for. As shown in our review, there is much variation in the type and number of variables included, which does not allow for a more in-depth comparison of the findings. It is crucial that future studies try to adjust their analyses for all key variables that might confound the relationship between risky decision-making and mental health. Indeed, adjusting for numerous confounders can decrease the likelihood of finding significant associations; nonetheless, the choice of confounders should be guided by the existing evidence, and not all studies included all the relevant demographic and socioeconomic characteristics (e.g., sex or gender, ethnicity, socioeconomic status) and the relevant psychological factors (e.g., intellectual ability and parental mental health) in their analyses. Therefore, we cannot exclude the possibility that, for those studies that under-adjusted their analyses, the associations might actually be non-significant. Finally, the significance of the findings might depend on which specific mental health or decision-making domain was analysed, thus highlighting the importance of exploring several specific domains to obtain more precise and realistic estimates. In fact, as shown by the results of this review, internalising and externalising symptoms were not associated with all possible aspects of risky decision-making. This is particularly evident in studies that used the CGT to assess these different aspects. For instance, four longitudinal studies used data from the same birth cohort and the significance of the results varied greatly depending on which decision-making aspects and which internalising and/or externalising symptoms were investigated [39, 53–55]. Taken together, these points highlight the need for more research examining the associations between risky decision-making and mental-ill health in the general youth population, as well as adopting a more methodical approach to establish a consensus on potential confounding factors. Moreover, given the conceptual complexity of risky-decision-making, it might be necessary to prioritise the use of gambling tasks that measure several aspects of risky decision-making rather than tasks assessing decision making more crudely.

This review presents with some limitations. First, despite the decision not to limit it to any particular country, the vast majority of the retrieved studies were conducted in Western countries, thus limiting the generalisability of the results to different cultures. Given that gambling tasks such as the IGT are used cross-culturally [61], the number of studies included in this review appears to be rather small. Second, due to the cross-sectional nature of some of the studies, it was not always possible to identify the direction of some of the relationships, particularly with regard to externalising symptoms. More longitudinal research is needed to understand the temporal order of these associations. Third, we decided to limit the review to gambling tasks only, meaning that other measures of risky decision-making might have been overlooked. Gambling tasks are widely used to assess various aspects of decision-making, however, future reviews might want to consider other aspects of reward processing, too, to obtain a more holistic overview of reward processing in the development of mental health problems. Relatedly, gambling tasks are susceptible to some validity and reliability issues. For instance, it has been suggested that the IGT might not be suitable to assess individual differences in risky decision-making due to low retest reliability and validity because of its task specificity, meaning that it might not be considered a general measure of risky decision-making [62]. This ultimately suggests that the findings should be replicated using other measures. Finally, given that this is a scoping review and the aim was to narratively describe the existing evidence in an explorative manner, the quality of the retrieved studies was not assessed. As a result of the exploratory nature of this review, our findings should not be used to directly inform clinical guidance or policy practice, but rather as a descriptive summary of the current evidence on this topic and the gaps in this field. Nonetheless, some recommendations are possible. For example, we recommend that specific aspects of both mental health problems and risky decision-making are analysed in order to better identify which components of these constructs are linked. Moreover, we recommend that future studies should investigate these relationships prospectively as well as, wherever possible, adopt intensive longitudinal designs in order to promptly identify changes and variations that are likely to occur at different developmental stages. Finally, we recommend that sociodemographic factors are seriously considered. For instance, stratifying the analyses by sex or gender could help pinpoint whether we can expect to see distinct patterns in the relationship between mental health and risky decision-making based on these differences, in turn allowing us to tailor interventions accordingly.

## Conclusions

In conclusion, we provide an overview of the current literature on the relationship between risky decision-making, measured using gambling tasks, and mental ill-health in the general youth population. Overall, most associations appear to lack statistical significance; however, some evidence of association exists, particularly with regard to hyperactivity. Further research in this area is warranted. This review also highlighted the need for future research to carefully consider confounder adjustment, as well as employ longitudinal and experimental designs to untangle temporal and causal relations. Furthermore, more studies should try to consider developmental differences (e.g., between children and adolescents) carefully. Moreover, different types of internalising and externalising symptoms and different domains of risky decision-making should be considered to ensure a better understanding of the relationship between risky decision-making and youth mental health. Finally, there may be merit in explicitly considering the role of sex and gender in this relationship.

### Supplementary Information

Supplementary Material 1.

## Data Availability

All data generated and/or analysed during this study are included in this published article [and its supplementary information files].

## Abbreviations

ADHD: Attention deficit hyperactivity disorder
BART: Balloon analogue risk task
CGT: Cambridge gambling task
IGT: Iowa gambling task
PGT: Preschool gambling task
SDQ: Strengths and difficulties questionnaire
