# Supplementary material for: The relationship between mental health and risky decision-making in children and adolescents: a scoping review
Source: BMC Psychiatry. 2024 Jun 5;24:424. doi: 10.1186/s12888-024-05850-9 (PMC11154980; doi:10.1186/s12888-024-05850-9)
Supplement: Supplementary file 1 — Supplementary Material 1. [file 12888_2024_5850_MOESM1_ESM.docx]

**Supplementary material**

Francesca Bentivegna, Efstathios Papachristou, Eirini Flouri

# The relationship between mental health and risky decision-making in children and adolescents: A scoping review.

Department of Psychology and Human Development, UCL Institute of Education, University College London, London, UK

**Table of Contents**

[Table S1. Description of gambling task (and different versions) used to assess risky decision-making. 2](#_Toc131274938)

[Table S2. Frequency and type of measures of gambling task and internalising and externalising symptoms. 4](#_Toc131274939)

[Preferred Reporting Items for Systematic reviews and Meta-Analyses extension for Scoping Reviews (PRISMA-ScR) Checklist 5](#_Toc131274940)

[MEDLINE search strategy of research articles investigating the association between internalising and/or externalising symptoms and risky decision-making measured using a gambling task in childhood and/or adolescence in the general population. 7](#_Toc131274941)

# Table S1. Description of gambling task (and different versions) used to assess risky decision-making.

| *Gambling task type (N studies)* | *Description of original task* | *Name of different versions used and description (N studies)* |
| --- | --- | --- |
| **Cambridge Gambling Task, CGT (9)** | A gambling task from the Cambridge Neuropsychological Test Automated Battery (CANTAB) which assesses various aspects of decision-making (Rogers et al., 1999). An interviewer guides the participant through the task while following a script and explains the structure of the assessment before the start of the trials. Participants sit in front of a computer screen were ten boxes, either red or blue, are displayed. They are told that a yellow token is hidden in one of the boxes. The task’s aim is to correctly guess in which box, red or blue, the token is hidden There are five stages and each comprises several blocks of trials. In the first stage (decision-making stage), participants have to make a decision as to whether the token is hidden in the red or the blue box. In the next stages (gambling stages) the participants are given 100 points and are asked to bet a proportion of these points. The aim is to win as many points as they can. They gain points by making correct bets, while they lose points in incorrect bets. The current bet value is displayed in a circle in the centre of the screen and can either incrementally increase or decrease depending on the task. The CGT produces six outcomes. *Delay aversion* is the difference in percentage bet in conditions where the bet value incrementally increases and in conditions where it incrementally decreases, and it reflects whether participants are prepared to wait in order to place a higher or lower bet; *deliberation time* is a measure of pre-motor processing and movement time and corresponds to the mean time taken (measured in milliseconds) to make a colour box response after the decision-making information has been presented; *risk-taking* is the mean proportion of points bet on trials where the most probable colour response is made, with more risk-taking indicating a higher sensitivity to reward/lower sensitivity to punishment; *risk adjustment* is the tendency to bet more points when the likelihood of correctly guessing where the token is hidden is high, i.e. when most boxes are either red or blue, compared to when that likelihood is low; *quality of decision-making* is the mean proportion of trials where the child bet on the most likely outcome, that is when the correct coloured box is chosen; finally, *overall proportion bet* is the mean proportion of points that are gambled across all trials. | Similar versions of the CGT can be shorter, or only a few outcome measures can be considered, or the ratios at which the ten boxes are presented on the screen can be amended. |
| **Balloon Analogue Risk Task, BART (4)** | A gambling task which measures risk-taking behaviour and where participants inflate balloons to earn money (Lejuez et al., 2007). Participants receive instructions prior to the beginning of the trial. They have to pump the balloon up by clicking a button, and the more they click the more the balloon inflate, meaning that they can earn more money. However, after a certain threshold (which the participants do not know), the balloon will explode, and all the money is lost. Participants can decide to stop inflating the balloon and collect the money, which then goes to a ‘permanent’ bank visible on the screen. More pumps correspond to a greater reward, but also to a higher chance to lose. Information related to both participants’ initial responses and the degree to which they adjust their responses over several trials (usually 15–30) is collected. The outcome measures are the number of explosions and the number of pumps adjusted for the number of balloons that did not explode (adjusted average of pumps). The BART models “real world” risk behaviour where the chance of reward or loss are balanced. | **Balloon Analogue Risk Task-Youth, BART-Y (2):** Same as original BART, but adapted for children, e.g. they earn points rather than money. |
| **Iowa Gambling Task, IGT (2)** | A gambling task that measures decision-making where participants need to choose one out of four decks of cards (Bechara et al., 1994). The original version was not computerised, however, it is now more common to perform the task in front of a screen. The decks are different in terms of the amount of money that participants can gain and the amount of money that they can lose, which can happen more or less frequently. There are both advantageous and disadvantageous decks. Two decks contain a higher immediate reward but also a higher loss, meaning that the long-term gain is smaller, while in the other two decks the immediate reward is lower but so is the loss. Participants are given the instructions prior to the start of the task, and they are told that the aim is to gain as much as money as possible. Participants are tested on their capacity to adapt and adjust their decisions over a certain number of trials (~100). Participants can also switch between decks of cards so that they can maximise their long-term rewards. | **Children’s Gambling Task (2):** A developmentally appropriate analogue of the IGT to assess decision-making where children receive candies or stickers rather than money (Kerr & Zelazo, 2004). Two decks of 50 cards each display either happy (win) or sad faces (loss). One deck has more rewards per trial, but due to occasional large losses, is disadvantageous across trials. The opposite happens with the second deck. Children can gain the same number of rewards in both decks but the number of losses varies across trials, which are divided into five blocks of ten trials each. Then, difference scores are calculated for each block, that is the proportion of advantageous choices minus the proportion of disadvantageous choices per block. |
|  |  | **Preschool Gambling Task, PGT (1):** A decision-making task inspired by the Child’s Gambling Task (Kerr & Zelazo, 2004), but amended to promote better motivation and attention to long-term outcomes. The PGT comprised two decks of 50 cards, one advantageous (attached to the image of a chick) and one disadvantageous (attached to the image of a giraffe). Children are told that they are going to play a card game, and that the goal is to win as many rewards (stickers) as possible. Children can win or lose stickers that can be used to mark their progress on a magnetic house with 40 stairs. They were then tested to see if they could identify the advantageous deck and explain the rationale behind their decision, and they are given points depending on whether they get one or both answers right. |
| **Balloon Context Task (0)** | A reward-feedback gambling task where balloons with different colours are displayed on a screen along a centred line, and participants have to use a four-option pad to select the balloon that they believe contains the monetary reward (Crowley et al., 2009; Holroyd et al., 2003). Participants receive a feedback at the end of each trial (~288; four blocks of trials with practice trials) letting them know whether they gained or lost money depending on their choice. The waiting period to receive the feedback (which is random) varied from short delay interval to long delay interval, thus creating four conditions: reward-short delay, reward-long delay, punishment-short delay, and punishment-long delay). The presentation of the balloon is random, meaning that no pattern can be established, however, participants are told that some people are capable of figuring out the patterns. The amount of money gained is visible on the screen and participants can make choices at their own pace. | **Money Maker Task (1):** A reward-feedback gambling task based on the Balloon Context Task which can be adapted to have more or less trials compared to the original version. |

# Table S2. Frequency and type of measures of gambling task and internalising and externalising symptoms.

|  | *Psychometric measure used*  *(N studies)* | *Type of measure* |
| --- | --- | --- |
| **Gambling task** | Cambridge Gambling Task, CGT (9) | Computerised assessment |
|  | Balloon Analogue Risk Task, BART (4) | Computerised assessment |
|  | Balloon Analogue Risk Task-Youth, BART-Y (2) | Computerised assessment |
|  | Iowa Gambling Task, IGT (2) | Computerised assessment |
|  | Children’s Gambling Task (2) | Card game |
|  | Preschool Gambling Task, PGT (1) | Card game |
|  | Money Maker Task (1) | Computerised assessment |
| **Internalising/externalising symptoms** | Strengths and Difficulties Questionnaire, SDQ (6) | Questionnaire |
|  | Short Mood and Feelings Questionnaire, sMFQ (2) | Questionnaire |
|  | Peer-reports (2) | Questionnaire |
|  | Problem Behaviour at School Interview, short version (2) | Questionnaire |
|  | Externalizing Disorder Inventory, EDI (1) | Questionnaire |
|  | Dysfunctional Attitudes Scale for Children, DASC (1) | Questionnaire |
|  | Abbreviated Social Phobia and Anxiety Inventory, SPAI-23 (1) | Questionnaire |
|  | Revised Child Anxiety and Depression Scale (1) | Questionnaire |
|  | Preschool Proactive and Reactive Aggression Scale (1) | Questionnaire |
|  | Depression Anxiety Stress Scale, DASS (1) | Questionnaire |
|  | Adolescent Behavior Assessment System for Children, Second ed, BASC-2 (1) | Questionnaire |
|  | Substance Use Risk Profile Scale, SURPS (1) | Questionnaire |
|  | Achenbach Child Behaviour Checklist (1) | Questionnaire |
|  | Youth Self Report of the Achenbach System of Empirically Based Assessment (1) | Questionnaire |
|  | Child Symptom Inventory-4, CSI-4 (1) | Questionnaire |
|  | Kinder Angst Test II, KAT II (1) | Questionnaire |
|  | Frequency of behaviours (1) | Questionnaire |

# Preferred Reporting Items for Systematic reviews and Meta-Analyses extension for Scoping Reviews (PRISMA-ScR) Checklist

| **SECTION** | **ITEM** | **PRISMA-ScR CHECKLIST ITEM** | **REPORTED ON PAGE #** |
| --- | --- | --- | --- |
| **TITLE** | | | |
| Title | 1 | Identify the report as a scoping review. | 1 |
| **ABSTRACT** | | | |
| Structured summary | 2 | Provide a structured summary that includes (as applicable): background, objectives, eligibility criteria, sources of evidence, charting methods, results, and conclusions that relate to the review questions and objectives. | 2 |
| **INTRODUCTION** | | | |
| Rationale | 3 | Describe the rationale for the review in the context of what is already known. Explain why the review questions/objectives lend themselves to a scoping review approach. | 3,4 |
| Objectives | 4 | Provide an explicit statement of the questions and objectives being addressed with reference to their key elements (e.g., population or participants, concepts, and context) or other relevant key elements used to conceptualize the review questions and/or objectives. | 4,5 |
| **METHODS** | | | |
| Protocol and registration | 5 | Indicate whether a review protocol exists; state if and where it can be accessed (e.g., a Web address); and if available, provide registration information, including the registration number. | 5 |
| Eligibility criteria | 6 | Specify characteristics of the sources of evidence used as eligibility criteria (e.g., years considered, language, and publication status), and provide a rationale. | 6,7 |
| Information sources* | 7 | Describe all information sources in the search (e.g., databases with dates of coverage and contact with authors to identify additional sources), as well as the date the most recent search was executed. | 5,6 |
| Search | 8 | Present the full electronic search strategy for at least 1 database, including any limits used, such that it could be repeated. | Supplementary material doc |
| Selection of sources of evidence† | 9 | State the process for selecting sources of evidence (i.e., screening and eligibility) included in the scoping review. | 5-7 |
| Data charting process‡ | 10 | Describe the methods of charting data from the included sources of evidence (e.g., calibrated forms or forms that have been tested by the team before their use, and whether data charting was done independently or in duplicate) and any processes for obtaining and confirming data from investigators. | 7,8 |
| Data items | 11 | List and define all variables for which data were sought and any assumptions and simplifications made. | 6,7 |
| Critical appraisal of individual sources of evidence§ | 12 | If done, provide a rationale for conducting a critical appraisal of included sources of evidence; describe the methods used and how this information was used in any data synthesis (if appropriate). | N/A |
| Synthesis of results | 13 | Describe the methods of handling and summarizing the data that were charted. | 7,8 |
| **RESULTS** | | | |
| Selection of sources of evidence | 14 | Give numbers of sources of evidence screened, assessed for eligibility, and included in the review, with reasons for exclusions at each stage, ideally using a flow diagram. | 8; Figure 1 doc |
| Characteristics of sources of evidence | 15 | For each source of evidence, present characteristics for which data were charted and provide the citations. | 8-10; Figure 1 doc, Table 1 |
| Critical appraisal within sources of evidence | 16 | If done, present data on critical appraisal of included sources of evidence (see item 12). | N/A |
| Results of individual sources of evidence | 17 | For each included source of evidence, present the relevant data that were charted that relate to the review questions and objectives. | 10-12 |
| Synthesis of results | 18 | Summarize and/or present the charting results as they relate to the review questions and objectives. | 12-16 |
| **DISCUSSION** | | | |
| Summary of evidence | 19 | Summarize the main results (including an overview of concepts, themes, and types of evidence available), link to the review questions and objectives, and consider the relevance to key groups. | 16-19 |
| Limitations | 20 | Discuss the limitations of the scoping review process. | 20,21 |
| Conclusions | 21 | Provide a general interpretation of the results with respect to the review questions and objectives, as well as potential implications and/or next steps. | 21 |
| **FUNDING** | | | |
| Funding | 22 | Describe sources of funding for the included sources of evidence, as well as sources of funding for the scoping review. Describe the role of the funders of the scoping review. | 22 |

JBI = Joanna Briggs Institute; PRISMA-ScR = Preferred Reporting Items for Systematic reviews and Meta-Analyses extension for Scoping Reviews.

* Where *sources of evidence* (see second footnote) are compiled from, such as bibliographic databases, social media platforms, and Web sites.

† A more inclusive/heterogeneous term used to account for the different types of evidence or data sources (e.g., quantitative and/or qualitative research, expert opinion, and policy documents) that may be eligible in a scoping review as opposed to only studies. This is not to be confused with *information sources* (see first footnote).

‡ The frameworks by Arksey and O’Malley (6) and Levac and colleagues (7) and the JBI guidance (4, 5) refer to the process of data extraction in a scoping review as data charting*.*

§ The process of systematically examining research evidence to assess its validity, results, and relevance before using it to inform a decision. This term is used for items 12 and 19 instead of "risk of bias" (which is more applicable to systematic reviews of interventions) to include and acknowledge the various sources of evidence that may be used in a scoping review (e.g., quantitative and/or qualitative research, expert opinion, and policy document).

*From:* Tricco AC, Lillie E, Zarin W, O'Brien KK, Colquhoun H, Levac D, et al. PRISMA Extension for Scoping Reviews (PRISMAScR): Checklist and Explanation. Ann Intern Med. 2018;169:467–473. [doi: 10.7326/M18-0850](http://annals.org/aim/fullarticle/2700389/prisma-extension-scoping-reviews-prisma-scr-checklist-explanation).

# MEDLINE search strategy of research articles investigating the association between internalising and/or externalising symptoms and risky decision-making measured using a gambling task in childhood and/or adolescence in the general population.

Database: Ovid MEDLINE(R) ALL <1946 to March 04, 2022>

Search Strategy:

--------------------------------------------------------------------------------

1 Affective Symptoms/ep, pa, px [Epidemiology, Pathology, Psychology] (6441)

2 psychological distress*.tw,ab,ti. (23585)

3 internalis*.tw,ab,ti. (3731)

4 internaliz*.tw,ab,ti. (64912)

5 emotional problem*.ab,ti. (5203)

6 emotional issue*.ab,ti. (542)

7 emotional symptom*.ab,ti. (1983)

8 exp Depression/ (138144)

9 depress*.ab,ti. (510755)

10 depress* symptom*.tw,ab,ti. (68952)

11 exp Anxiety/ (100572)

12 anxiety.tw,ab,ti. (225048)

13 anxiety symptom*.ab,ti. (12458)

14 peer problem*.ab,ti. (705)

15 exp Child Behavior/ (26107)

16 child behavio?r*.ab,ti. (8360)

17 exp Problem Behavior/ (3258)

18 problem behavio?r*.ab,ti. (5190)

19 externalis*.tw,ab,ti. (1139)

20 externaliz*.tw,ab,ti. (14671)

21 behavio* problem*.ab,ti. (17954)

22 behavio* issue*.ab,ti. (968)

23 behavio* symptom*.ab,ti. (5362)

24 conduct.tw,ab,ti. (95468)

25 antisocial behavio?r*.tw,ab,ti. (4217)

26 hyper*.tw,ab,ti. (1602700)

27 inattent*.ab,ti. (8212)

28 attention deficit*.ab,ti. (32978)

29 exp Mental Health/ (51010)

30 mental health.tw,ab,ti. (176834)

31 mental health problem*.ab,ti. (16412)

32 mental health issue*.ab,ti. (4172)

33 mental health symptom*.ab,ti. (2832)

34 mental health disorder*.ab,ti. (4981)

35 exp Reward/ (24748)

36 reward*.tw,ab,ti. (59987)

37 reward* process*.tw. (2895)

38 reward sensitiv*.tw. (906)

39 reward hypersensi*.tw. (20)

40 reward hyposensi*.tw. (5)

41 punishment sensitiv*.tw. (189)

42 punishment hypersensi*.tw. (1)

43 punishment hyposensi*.tw. (2)

44 decision making.ab,ti. (160243)

45 (mak* adj3 decision*).ab,ti. (206366)

46 delay discount*.ab,ti. (1729)

47 exp Risk-Taking/ (34543)

48 risk tak*.ab,ti. (7819)

49 (tak* adj3 risk*).ab,ti. (12962)

50 gambling task*.mp. (1977)

51 cambridge gambling task*.tw,ab,ti. (83)

52 CGT.tw. (919)

53 iowa gambling task*.tw,ab,ti. (1171)

54 IGT.tw. (5206)

55 balloon analogue risk task*.tw,ab,ti. (279)

56 BAGT.tw. (6)

57 exp Child/ (2054131)

58 (child* or stepchild* or step-child* or kid or kids or girl or girls or boy or boys or teen* or youth* or youngster* or adolescent* or adolescence or preschool* or pre-school* or kindergarten* or school* or reception or elementary or primary school* or middle school* or juvenile* or minors or p?ediatric* or PICU).ti,ab. (2250070)

59 Adolescent/ (2161904)

60 (teen* or youth* or adolescen* or juvenile* or youngster* or first-grader* or second-grader* or third-grader* or fourth-grader* or fifth-grader* or sixth-grader* or seventh-grader* or highschool* or ((secondary or high*) adj2 (school* or education))).ti,ab. (521438)

61 (meta-analysis or review literature).sh. (154127)

62 meta-analy$.tw. (225678)

63 metaanal$.tw. (2620)

64 (systematic$ adj4 (review$ or overview$)).tw. (256681)

65 meta-analysis.pt. (154127)

66 review.pt. (2947004)

67 letter.pt. (1171274)

68 historical article.pt. (367877)

69 review.ti. (589718)

70 61 or 62 or 63 or 64 or 65 or 66 or 69 (3311106)

71 67 or 68 (1531428)

72 70 not 71 (3271450)

73 exp Animals/ (25193846)

74 Humans/ (20226411)

75 73 not 74 (4967435)

76 1 or 2 or 3 or 4 or 5 or 6 or 7 or 8 or 9 or 10 or 11 or 12 or 13 or 14 or 15 or 16 or 17 or 18 or 19 or 20 or 21 or 22 or 23 or 24 or 25 or 26 or 27 or 28 or 29 or 30 or 31 or 32 or 33 or 34 (2563085)

77 35 or 36 or 37 or 38 or 39 or 40 or 41 or 42 or 43 or 44 or 45 or 46 or 47 or 48 or 49 (306739)

78 50 or 51 or 52 or 53 or 54 or 55 or 56 (7584)

79 57 or 58 or 59 or 60 (4064922)

80 76 and 77 and 78 and 79 (164)

81 80 not 72 (163)

82 81 not 75 (162)

83 limit 82 to english language (159)

***************************
